# Description and complete mitochondrial genome of *Atkinsoniella zizhongi* sp. nov. (Hemiptera: Cicadellidae: Cicadellinae) from China and its phylogenetic implications

**DOI:** 10.7717/peerj.14026

**Published:** 2022-09-28

**Authors:** Yan Jiang, Hao-Xi Li, Xiao-Fei Yu, Mao-Fa Yang

**Affiliations:** 1 Guizhou University, Institute of Entomology, Guiyang, Guizhou Province, China; 2 Guizhou Provincial Key Laboratory for Agricultural Pest Management of the Mountainous Region, Guiyang, Guizhou Province, China; 3 Guizhou University, College of Tobacco Sciences, Guiyang, Guizhou Province, China

**Keywords:** Hemiptera, Cicadellinae, New species, Mitogenome, Phylogenetics

## Abstract

A new species, *Atkinsoniella zizhongi* sp. nov. of the subfamily Cicadellinae, was described and illustrated from China. The new species is similar to *A*. *nigrominiatula* (Jacobi, 1944), *A*. *limba* Kuoh, 1991, *A*. *dormana* Li, 1992, *A*. *peaka* Yang, Meng *et* Li, 2017, and *A*. *divaricata* Yang, Meng *et* Li, 2017. But the characteristics of aedeagus and pygofer process can be used to distinguish them easily. The complete mitochondrial genome of the paratype was sequenced and assembled. The mitogenome of *A*. *zizhongi* sp. nov. was 16,483 bp in length, with an A+T content of 75.9%, containing 37 typical genes and a control region (CR). The gene order was consistent with the inferred insect ancestral mitochondrial genome. All of the PCGs were determined to have the typical stop codon TAA or TAG, while COX2 and ND5 ended with incomplete termination codons T and TA, respectively. In addition, phylogenetic trees were reconstructed based on PCGs and rRNAs using both the maximum likelihood (ML) and Bayesian inference (BI) methods. The results showed that the intergeneric and interspecific relationships within the subfamily Cicadellinae were completely consistent in all of the phylogenetic trees, except that the different interspecific relationships within the genus *Bothrogonia* were detected in the ML analysis based on the amino acid sequences. This study enriches the species diversity of Cicadellinae and further promotes research on its phylogeny.

## Introduction

Cicadellidae is one of the most diverse families in Hemiptera, with over 23,000 described species distributed worldwide (Dietrich, 2004, 2005). Cicadellidae species are commonly known as leafhoppers and have variable color patterns and sizes. Cicadellinae is a large and diverse subfamily within the family Cicadellidae, comprising approximately 2,547 known species in approximately 333 genera widely distributed in all zoogeographic regions of the world, with 259 species in 23 genera recorded in China after *Mileewa*, *Ujna*, and *Processina* were placed into the subfamily Mileewinae (Young, 1968; Linnavuori & Delong, 1977; Feng & Zhang, 2017; Yang, Meng & Li, 2017; Naveed & Zhang, 2018). All known Cicadellinae insects are xylem feeders, with lengths ranging from 4 to 19 mm. Some Cicadellinae species are of considerable economic importance, as they injure plants by directly feeding on sap in the xylem or indirectly transmitting phytopathogenic bacteria and plant viruses (Hopkins & Purcell, 2002; Redak et al., 2004; Cornara et al., 2019; Krugner et al., 2019; Kleina et al., 2020). The diversity of Cicadellinae and the similar morphological characteristics among some species make their accurate identification difficult at the species level. In particular, some species within the genera *Atkinsoniella*, *Bothrogonia* and *Kolla* are very commonly found with similar morphological characteristics, resulting in they can’t be identified to the species level without male genitalia. In this situation, many female specimens with similar colors and markings cannot be matched with the corresponding male specimens; they can only be identified to the genus level. Therefore, in addition to the traditional morphological classification, molecular techniques are needed to help us identify the species more accurately and understand their phylogenetic implications.

The insect mitochondrial genome (mitogenome) is a circular DNA molecule with a length typically ranging from 15 to 18 kb (Cameron, 2014). It contains 13 protein-coding genes (PCGs), 22 transfer RNA genes (tRNAs), two ribosomal RNA genes (rRNAs), and a large noncoding region, which is termed the control region and alternatively called the A+T–rich region (CR) (Cameron, 2014). The mitogenome has the characteristics of a simple and stable structure, high genome copy numbers, maternal inheritance, conserved gene components, and a relatively high evolutionary rate (Wolstenholme, 1992; Boore, 1999; Curole & Kocher, 1999; Cameron, 2014). It has been widely used for studying the evolutionary genetics and phylogenetic relationships of various taxonomic levels of insects (Cameron, 2014; Li et al., 2014, 2017; Liu et al., 2019; Chen et al., 2020a). To date, only 37 mitochondrial genomes of subfamily Cicadellinae have been submitted to GenBank, but some taxa from the New World may be misidentified. The published mitogenomes are scarce for the subfamily Cicadellinae, which contains such rich species. And there are few studies on the mitogenomic phylogeny within Cicadellinae. Only Jiang et al. (2022) conducted the intergeneric and interspecific relationships within Cicadellinae based on all the available Cicadellinae mitogenomes. The others mainly focus on the relationships among the subfamilies of Cicadellidae, with few Cicadellinae sequences were cited. Therefore, it is necessary to obtain more mitogenomes to elucidate the relationships within Cicadellinae.

In the present study, a new species of *Atkinsoniella* was described and illustrated from China. The complete mitogenome sequence of the new species was assembled and annotated to further understand the characteristics of Cicadellinae species. Furthermore, phylogenetic analyses were constructed based on the mitogenome sequences of Cicadellinae taxa to clarify the relationships within this group. We expect this study to be valuable for phylogenetic studies of the subfamily Cicadellinae and higher taxonomic categories.

## Materials and Methods

### Sample collection and description of the new species

Specimens were obtained by a sweeping net. The collections were approved by the following institution: Yingshan Agricultural Bureau, Hubei Province, China; Shennongjia Scenic Area, Hubei Province, China; Rongjiang Agricultural Bureau, Guizhou Province, China; Zhejiang Tianmu Mountain National Nature Reserve Bureau, Zhejiang Province, China. Habitus photographs were taken with a KEYENCE VHX-6000 digital camera. The length of the body was measured from the vertex to the apex of the forewings. The abdomen of the specimen was soaked in 10% NaOH solution, boiled for 1–3 min, rinsed with distilled water to remove any trace of NaOH, and transferred to glycerol for further dissection and photography. The male genitalia were photographed using a Nikon Eclipse Ni-E microscope. The holotype and all paratypes of the new species were permanently deposited at the Institute of Entomology, Guizhou University, Guiyang, China (GUGC). The morphological terminology follows Young (1986). The sample used to extract the total genomic DNA was collected from Tianmu Mountain, Zhejiang Province, China, immediately preserved in 100% ethanol and stored at −20 °C in the laboratory before DNA extraction.

The electronic version of this article in Portable Document Format (PDF) will represent a published work according to the International Commission on Zoological Nomenclature (ICZN), and hence the new names contained in the electronic version are effectively published under that Code from the electronic edition alone. This published work and the nomenclatural acts it contains have been registered in ZooBank, the online registration system for the ICZN. The ZooBank LSIDs (Life Science Identifiers) can be resolved and the associated information viewed through any standard web browser by appending the LSID to the prefix http://zoobank.org/. The LSID for this publication is: urn:lsid:zoobank.org:pub:5C835103-B7B9-499D-A48D-C128090D4011. The online version of this work is archived and available from the following digital repositories: PeerJ, PubMed Central SCIE and CLOCKSS.

### DNA library construction and sequencing

Total genomic DNA was extracted from the head and thorax muscle tissues of a single sample soaked in absolute ethanol using a DNeasy® Tissue Kit (Qiagen, Hilden, Germany) following the manufacturer’s instructions. The remaining wings and abdomen (including the male genitalia) of the tested samples were deposited at the Institute of Entomology, Guizhou University, Guiyang, China (GUGC) as vouchers for species identification. The obtained total genomic DNA was used for library preparation and next-generation sequencing (Illumina NovaSeq6000 platform, Berry Genomic, Beijing, China) with a paired-end 150 sequencing strategy. Clean sequencing data (6 Gb) were obtained and assembled using NOVOPlasty 2.7.2 (Dierckxsens, Mardulyn & Smits, 2017) with the *COX1* fragment of *A*. *xanthoabdomena* (GenBank accession number: ON428479) as the seed to start.

### Mitogenome annotation and characteristics analysis

The preliminary annotation of the sequences was determined by Mitoz 2.4-alpha (Meng et al., 2019) with the invertebrate mitochondrial genetic codes. The boundaries and secondary structures of the transfer RNA genes were reconfirmed and predicted by employing the MITOS2 web server (http://mitos2.bioinf.uni-leipzig.de/index.py) (Bernt et al., 2013) and the tRNAscan-SE search web server (Lowe & Chan, 2016). The 13 protein-coding genes (PCGs) were confirmed by finding the open reading frames (ORFs) with the invertebrate mitochondrial genetic code and aligning them with other published Cicadellidae mitogenomes using MAFFT 1.4.0 (Katoh & Standley, 2013) implemented in Geneious Prime 2022.1.1 (https://www.geneious.com) with the default parameters. The two ribosomal RNA genes (l-rRNA and s-rRNA) were adjusted according to the locations of the adjacent tRNA genes (trnL1 and trnV) and the alignment with the homologous rRNA genes of other published Cicadellidae mitogenomes. The visualization of the complete mitogenome structure was conducted by Geneious Prime 2022.1.1. MEGA 6.0 (Tamura et al., 2013) was employed to compute the nucleotide composition statistics and relative synonymous codon usage (RSCU) values of each PCG. Strand asymmetry was calculated manually based on the formulas AT skew = [A − T]/[A + T] and GC skew = [G − C]/[G + C] (Perna & Kocher, 1995). MEGA 6.0 was also used to calculate the Kimura 2-parameter genetic distances. The sliding window analysis (a sliding of 200 bp and a step size of 20 bp) and nonsynonymous (Ka) and synonymous (Ks) substitution ratios based on the 13 aligned PCGs were performed using DnaSP v6.12.03 (Rozas et al., 2017). The complete mitogenome of the new species was submitted to GenBank with the accession number ON457601.

### Phylogenetic analysis

In the phylogenetic analysis, the newly obtained mitogenome of *A*. *zizhongi* sp. nov., and 28 mitogenomes (all the available mitogenomes of Cicadellinae at present, except for some doubted taxa) from eight genera of subfamily Cicadellinae available in the GenBank database (https://www.ncbi.nlm.nih.gov/genbank/) were selected as the ingroup. *Mileewa margheritae* (MT483998) and *Parazyginella tiani* (NC_053918) from subfamilies Mileewinae and Typhlocybinae, respectively, were considered as the outgroup. Three concatenated datasets were used for phylogenetic analysis: (1) cds_faa: amino acid sequences of the PCGs; (2) cds12_fna: 1st and 2nd positions of the PCGs; (3) cds12_rrna: 1st and 2nd positions of the PCGs and two rRNAs. The 13 PCGs and two rRNAs were extracted using PhyloSuite 1.2.2 (Zhang et al., 2020). The PCGs and rRNAs were aligned by the MACSE (Ranwez et al., 2011) algorithm in PhyloSuite 1.2.2 with the invertebrate mitochondrial genetic code. The 2 rRNAs were aligned with MAFFT using the G-INS-I strategy. The gaps and ambiguous sites were removed from the alignments using the Gblocks (Castresana, 2000; Talavera & Castresana, 2007) algorithm in PhyloSuite 1.2.2 under the default settings. The 1st and 2nd positions of the PCGs were extracted from the aligned PCG datasets using MEGA 6.0 Alignments of each individual gene were concatenated using PhyloSuite 1.2.2. Maximum likelihood (ML) trees were reconstructed using IQ-TREE v.1.6.8 (Nguyen et al., 2015) under the optimal partitioning schemes and substitution models estimated by ModelFinder (Kalyaanamoorthy et al., 2017) implemented in the IQ-TREE package with the Bayesian information criterion (BIC). The branch support was estimated with 10,000 replicates of ultrafast bootstrap. Bayesian inference (BI) trees were reconstructed using MrBayes 3.2.6 (Ronquist & Huelsenbeck, 2003) under the best partitioning schemes and fitting substitution models determined in PartitionFinder v.2.1.1 (Lanfear et al., 2017) with the BIC criterion and greedy search algorithm. BI analysis using the default settings by four simultaneous Markov chains was run for 5–10 million generations in two independent runs, with sampling every 1,000 generations, and the initial 25% of samples were discarded as burn-in.

## Results and discussion

### Taxonomy

Genus *Atkinsoniella* Distant, 1908

Type species. *Atkinsoniella decisa* Distant, 1908

**Distribution.** Palaearctic, Oriental.

**New species**

***Atkinsoniella zizhongi* Jiang & Yang sp. nov.**

zoobank.org:act:AE46396A-3295-4284-9830-60FEB4398BF9

BioProject: PRJNA863844

SRA: PRJNA863844

(Figs. 1 and 2)

**Figure 1:**
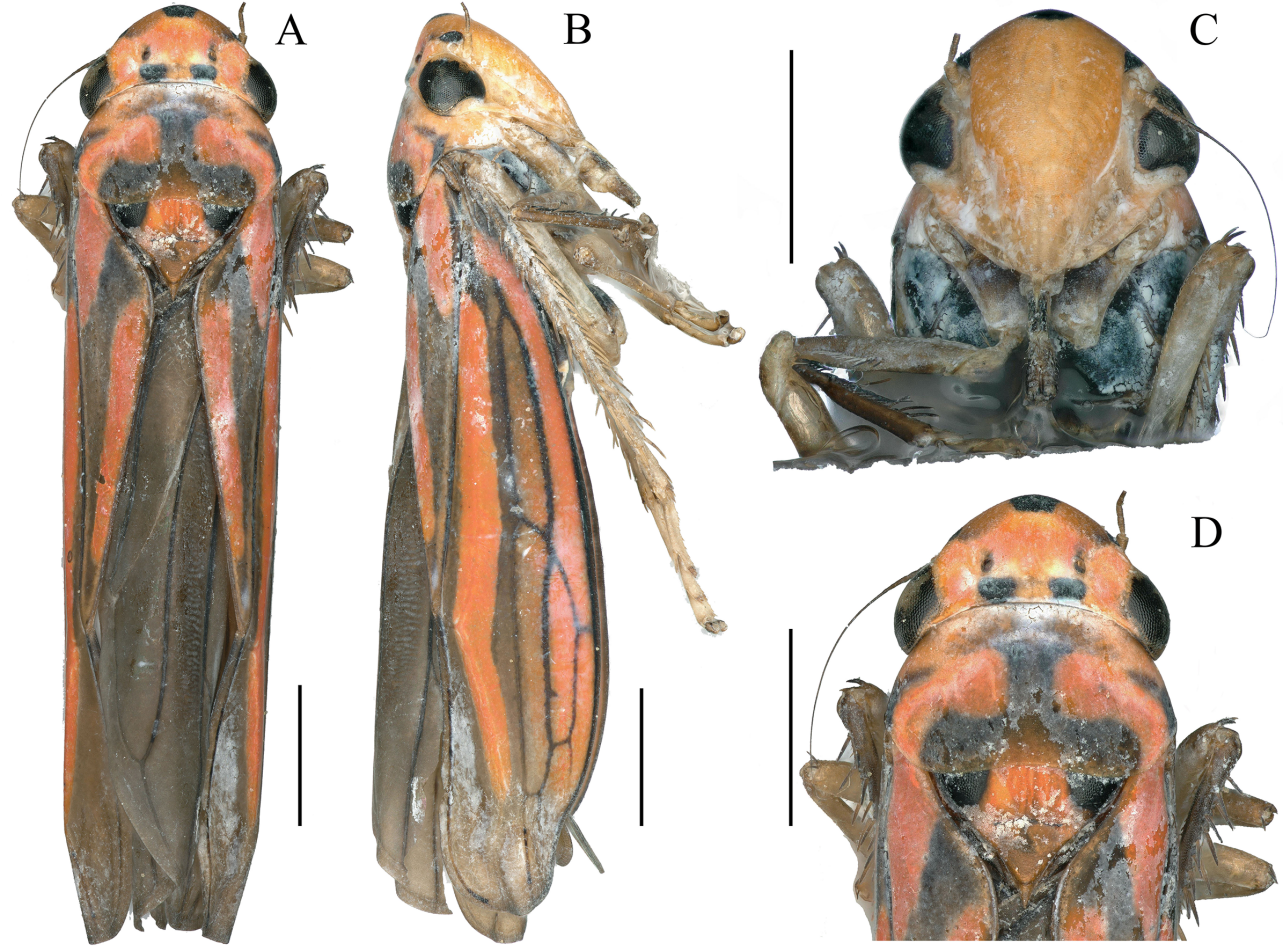
External features of *Atkinsoniella zizhongi* sp. nov., male holotype (total length 6.8 mm). (A) habitus, dorsal view; (B) habitus, lateral view; (C) face, anterior view; (D) head and pronotum, dorsal view. Scale lines = 1,000 μm.

**Figure 2:**
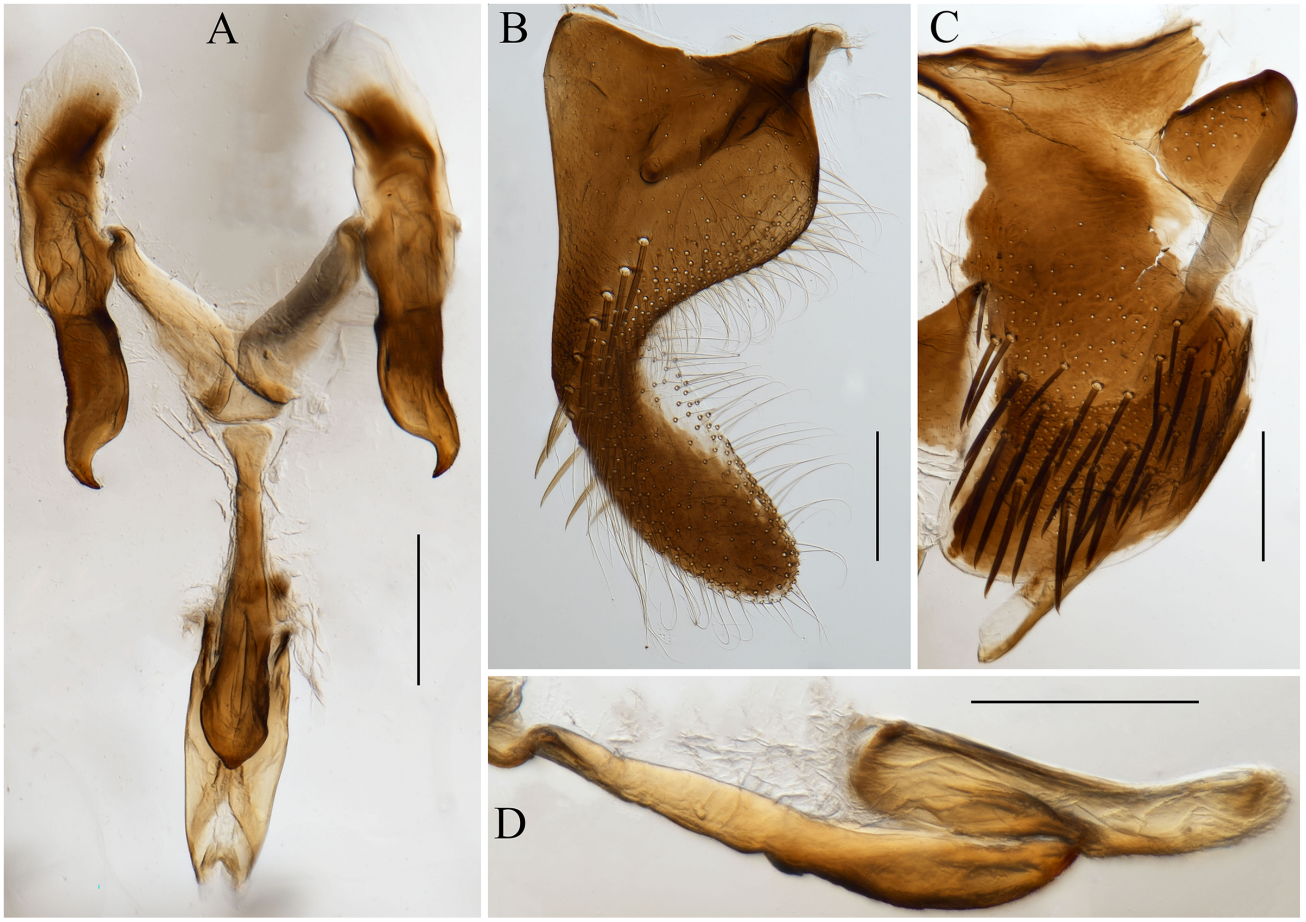
Male genitalia of *Atkinsoniella zizhongi* sp. nov. (A) Connective, style, aedeagus and paraphysis, ventral view; (B) subgenital plate, ventral view; (C) pygofer, lateral view; (D) aedeagus and paraphysis, lateral view. Scale lines = 200 μm.

**Length.** Male holotype 6.8 mm, male paratypes 6.5–6.8 mm (*n* = 6).

Dorsum tangerine; the center of the vertex with a large suborbicular black spot; one black spot beneath each ocellus at the basal margin of the crown; a small black spot in front of each ocellus in some individuals; eyes and ocelli dark brown; pronotum with a black “⊥” pattern markings and a pair of symmetrical black longitudinal spots at each lateral margin; scutellum with a large black spot at each angle near the pronotum and connected to the “⊥” pattern markings on the pronotum; forewing black–brown with four orange longitudinal stripes, two short in clavus, the other two long in corium, veins black; face reddish-yellow or red, each antennal ledge with zero or one black streak; mesopleuron, metapleuron, mesothethium, and metastethium black; legs brown or dark brown; abdomen in the ventral view black (Fig. 1).

Anterior margin of the crown broadly rounded and convex; the crown flat, but the lateral area of the ocellus concave; median length of the crown shorter than the interocular width; ocelli located behind the imaginary line between the anterior eye angles and the tip of the lateral clypeal suture, each ocellus closer to the adjacent eye angle than to another ocellus. Face with frons convex, muscle impressions distinct and some extend to the tip of crown; clypeal sulcus obscure medially. Thorax in dorsal view, with the pronotum slightly convex and the width almost equal to the transocular width of the head; posterior margin slightly concave medially; lateral margins slightly convergent anteriorly; dorsolateral carina not quite attaining the posterior margin of the eye. Mesonotum, with the scutellum convex before and behind the transverse depression; forewings with four apical cells, base of the second and third cells almost aligned transversely, or the base of the second cells more basal than that of the third cells transversely.

Male pygofer broadly rounded posteriorly, posterior margin convex dorsally, with macrosetae in the posterior half; pygofer process arising basiventrally on each side, and extending dorsolateral posteriorly of the pygofer, with the dorsad membranous transparent apically; subgenital plate in the ventral view, with a uniseriate row of macrosetae, long and short microsetae near the lateral margin and the entire ventral surface in the apical half; connective Y-shaped, extending posteriorly farther than the midlength of the style but not as far as the style apex, lateral arms tapered apically into a hook and manubrium short; style broad, with a hook inward at the apex; paraphysis longer than the aedeagus, acute apically and the ventral margin undulating medially, and dorsally articulating with the aedeagus apically; aedeagus concave at the base and tip in the ventral view, bent dorsally in the apical one-third portion, tip obtuse and the ventral margin concave medially in the lateral view (Fig. 2).

**Etymology**. This species is dedicated to Prof. Zi-Zhong Li, Institute of Entomology, Guizhou University, Guiyang, China, for his great contribution to the taxonomy of Cicadellidae.

**Material examined.** Holotype (GUGC-20220325-1a): ♂, Yingshan County, Hubei Province, China, 24 June 2014, coll. Zai-Hua Yang. Paratypes (GUGC-20220325-1b to 1g): 2 ♂♂, Shennongjia Scenic Area, Hubei Province, China, 18 July 2013, coll. Hu Li; 1 ♂, Rongjiang County, Guizhou Province, China, 25 July 2016, coll. Yan-Li Zheng, and Nian Gong; 2 ♂♂, Rongjiang County, Guizhou Province, China, 25 July 2016, coll. Yao-Wen Zhang, Ying-Jian Wang, and Yan-Li Zheng; 1 ♂, Tianmu Mountains, Zhejiang Province, China, 28 July 2018, coll. Li-Kun Zhong (the specimen used to extract the total genomic DNA).

**Remarks.** The new species is similar to *A*. *nigrominiatula* (Jacobi, 1944), *A*. *limba* Kuoh, 1991, *A*. *dormana* Li, 1992, *A*. *peaka* Yang, Meng *et* Li, 2017, and *A*. *divaricata* Yang, Meng *et* Li, 2017 but can be readily differentiated from the latter by the following characteristics: pygofer process not acute at the apex, and dorsad membranous transparent apically; aedeagus concave at the base and tip in the ventral view, bent dorsally on the apical one-third portion, tip obtuse and ventral margin concave medially in the lateral view.

### Genome organization and nucleotide composition

**Figure 3:**
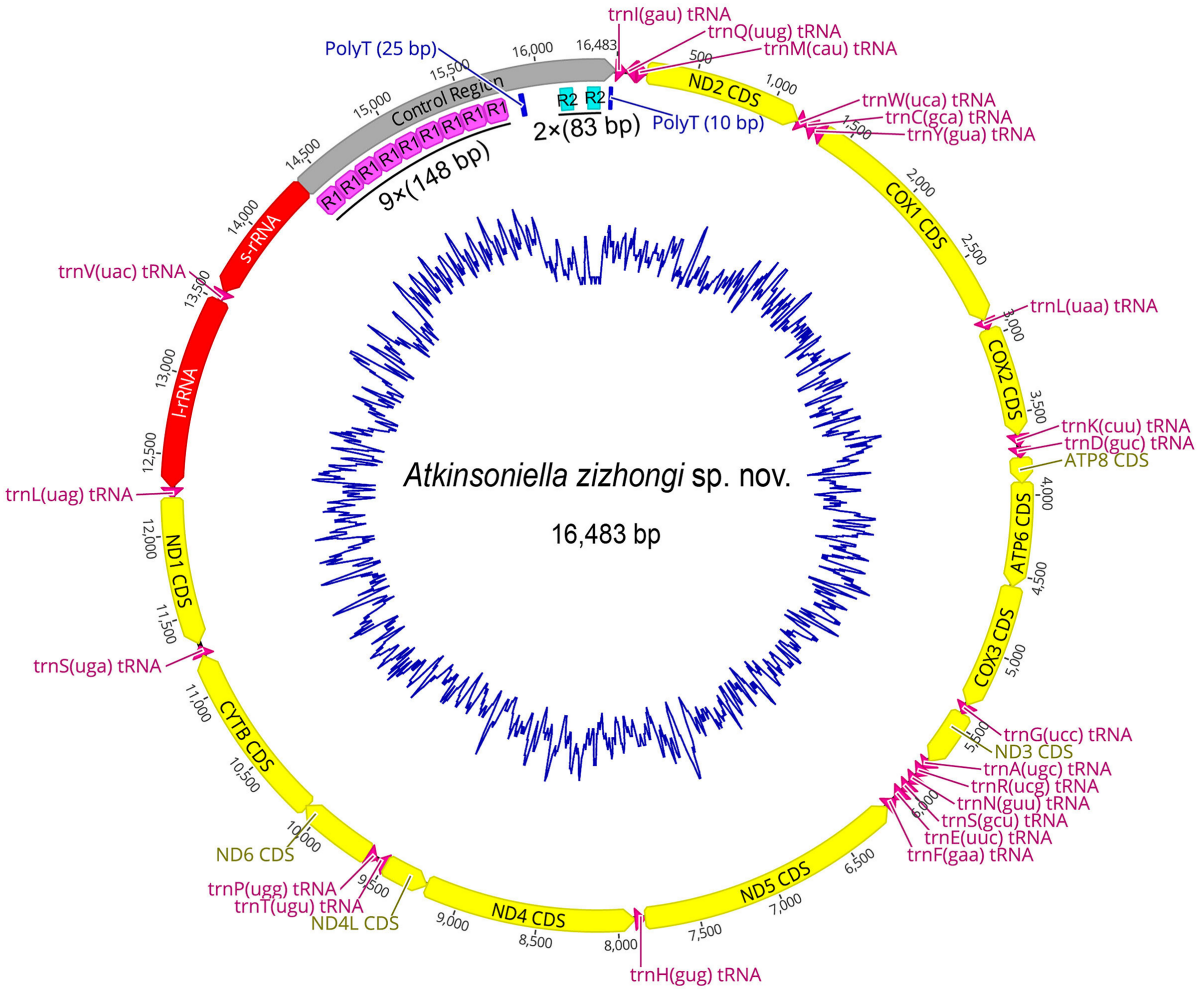
Circular map of the mitochondrial genome of *Atkinsoniella zizhongi* sp. nov. Protein-coding, transfer, and ribosomal RNA genes are shown with standard abbreviations. Gene orientations are indicated by arrow directions. Protein-coding genes, transfer RNA genes, two ribosomal RNA genes, and control region are shown in yellow, aubergine, red, and gray, respectively. R refers to repeat unit, with the number indicating the number of repeats. The blue blocks refer to the structures of poly (A) and poly (T).

The mitogenome of *A. zizhongi* sp. nov. was double-stranded circular with a length of 16,483 bp, containing 37 typical genes (13 PCGs, 22 tRNAs, and two rRNAs) and a control region (Fig. 3 and Table 1). The gene order was identical to the typical gene arrangement of the ancestral insect mitogenome (Cameron, 2014). Twenty-three genes (nine PCGs and 14 tRNAs) were encoded on the majority strand (J-strand), while the remaining 14 genes (four PCGs, eight tRNAs, and two rRNAs) were encoded on the minority strand (N-strand). A total of 49 bp overlaps across 16 locations were detected, and the longest 8 bp overlap was situated between the *trnW* and *trnC* genes. In addition, there were eight intergenic spacers, involving 30 bp in total, and the longest spacer sequence of 15 bp was located between *trnS2* and *ND1*. The overall nucleotide composition of the *A. zizhongi* sp. nov. mitogenome was A: 41.1%, T: 34.8%, C: 12.3%, and G: 11.8%, with significantly biased A and T nucleotides and a 75.9% A+T content (Table 2). The AT skew and GC skew were positive and negative, with values of 0.082 and −0.022, respectively, indicating that As and Cs were more abundant than Ts and Gs, which is consistent with the published complete Cicadellinae mitogenomes (Jiang et al., 2021).

**Table 1:** Organization of the *Atkinsoniella zizhongi* sp. nov. mitochondrial genome.

Name	Start	Stop	Size (bp)	Direction	Anticodon	Start codon	Stop codon	Intergenic nucleotides
*trnI*	1	63	63	J	GAU			−3
*trnQ*	61	128	68	N	UUG			3
*trnM*	132	200	69	J	CAU			0
*ND2*	201	1,172	972	J		ATT	TAA	−2
*trnW*	1,171	1,237	67	J	UCA			−8
*trnC*	1,230	1,290	61	N	GCA			0
*trnY*	1,291	1,354	64	N	GUA			1
*COX1*	1,356	2,891	1,536	J		ATG	TAA	1
*trnL*	2,893	2,955	63	J	UAA			0
*COX2*	2,956	3,634	679	J		ATT	T	0
*trnK*	3,635	3,705	71	J	CUU			3
*trnD*	3,709	3,776	68	J	GUC			0
*ATP8*	3,777	3,929	153	J		TTG	TAG	−7
*ATP6*	3,923	4,573	651	J		ATG	TAA	3
*COX3*	4,577	5,356	780	J		ATG	TAA	−1
*trnG*	5,356	5,418	63	J	UCC			−3
*ND3*	5,416	5,772	357	J		ATA	TAG	−2
*trnA*	5,771	5,831	61	J	UGC			0
*trnR*	5,832	5,899	68	J	UCG			−2
*trnN*	5,898	5,962	65	J	GUU			−1
*trnS1*	5,962	6,027	66	J	GCU			0
*trnE*	6,028	6,091	64	J	UUC			−1
*trnF*	6,091	6,155	65	N	GAA			−1
*ND5*	6,155	7,833	1,679	N		ATT	TA	−3
*trnH*	7,831	7,892	62	N	GUG			−1
*ND4*	7,892	9,211	1,320	N		ATA	TAA	−4
*ND4L*	9,208	9,489	282	N		ATG	TAA	2
*trnT*	9,492	9,553	62	J	UGU			0
*trnP*	9,554	9,617	64	N	UGG			2
*ND6*	9,620	10,108	489	J		ATT	TAA	−8
*CYTB*	10,101	11,237	1,137	J		ATG	TAG	−2
*trnS2*	11,236	11,299	64	J	UGA			15
*ND1*	11,315	12,232	918	N		ATT	TAA	0
*trnL*	12,233	12,295	63	N	UAG			0
*l-rRNA*	12,296	13,500	1,205	N				0
*trnV*	13,501	13,563	63	N	UAC			0
*s-rRNA*	13,564	14,373	810	N				0
control region	14,374	16,483	2,110					

**Table 2:** The nucleotide composition of *Atkinsoniella zizhongi* sp. nov. mitochondrial genome.

Regions	Length (bp)	T%	C%	A%	G%	A+T%	AT skew	GC skew
Whole genome	16,483	34.8	12.3	41.1	11.8	75.9	0.082	−0.022
PCGs	10,953	43.2	11.9	32.6	12.4	75.7	−0.140	0.022
1st codon position	3,650	35.8	11.3	35.4	17.5	71.2	−0.005	0.214
2nd codon position	3,650	47.8	17.4	21.3	13.5	69.1	−0.383	−0.125
3rd codon position	3,650	45.9	6.9	41.0	6.2	86.9	−0.057	−0.052
tRNAs	1,424	38.3	9.3	39.7	12.7	78.0	0.017	0.157
rRNAs	2,015	46.3	8.3	33.3	12.1	79.6	−0.162	0.187
Control region	2,110	34.6	9.8	37.1	18.5	71.7	0.035	0.308

### Protein-coding genes and codon usage

The mitogenome of *A. zizhongi* sp. nov. contained 13 PCGs in the typical order, with a total length of 10,953 bp, encoding 3,639 amino acids in total. All of the PCGs were initiated with the typical start codons ATD (ATA, ATT, or ATG) and terminated with the complete stop codon TAA or TAG, with the exception that *ATP8* was started with a TTG codon, and the truncated stop codons T and TA were detected in *COX2* and *ND5*, respectively. An incomplete termination codon is commonly observed in Hemiptera and metazoan mitogenomes (Su & Liang, 2018; Wang et al., 2018; Du, Dietrich & Dai, 2019; Zhang et al., 2019; Chen et al., 2020b). Four PCGs (*ND1*, *ND4*, *ND4 L*, and *ND5*) were encoded on the N-strand, and the other nine PCGs (*ND2*, *ND3*, *ND6*, *COX1*, *COX2*, *COX3*, *ATP6*, *ATP8*, *CYTB*) were encoded on the J-strand. The A+T content of the 13 PCGs was 75.7% (A: 32.6%, T: 43.2%, C: 11.9%, G: 12.4%), with a negative AT skew (−0.140) and a positive GC skew (0.022), which was consistent with the previously reported Cicadellinae (Jiang et al., 2021). Meanwhile, the AT content of the third codon position was higher than that of the first and second codon positions, with values of 86.9%, 71.2% and 69.1%, respectively (Table 2). The codon number and relative synonymous codon usage (RSCU) of the PCGs are summarized in Fig. S1, showing that the four amino acids with the highest frequency were phenylalanine, leucine, isoleucine, and methionine, and the most prevalent codons were UUU, UUA, AUU, and AUA, which were comprised of A and U. The others, such as CCG, GCC, CGC, AGG, AGC, CUG and UGC with rich G and C were the least utilized codons. Additionally, the RSCU of the PCGs showed that degenerate codons were biased to use more A or T than G or C at the third codon position.

### Transfer RNA and ribosomal RNA

**Figure 4:**
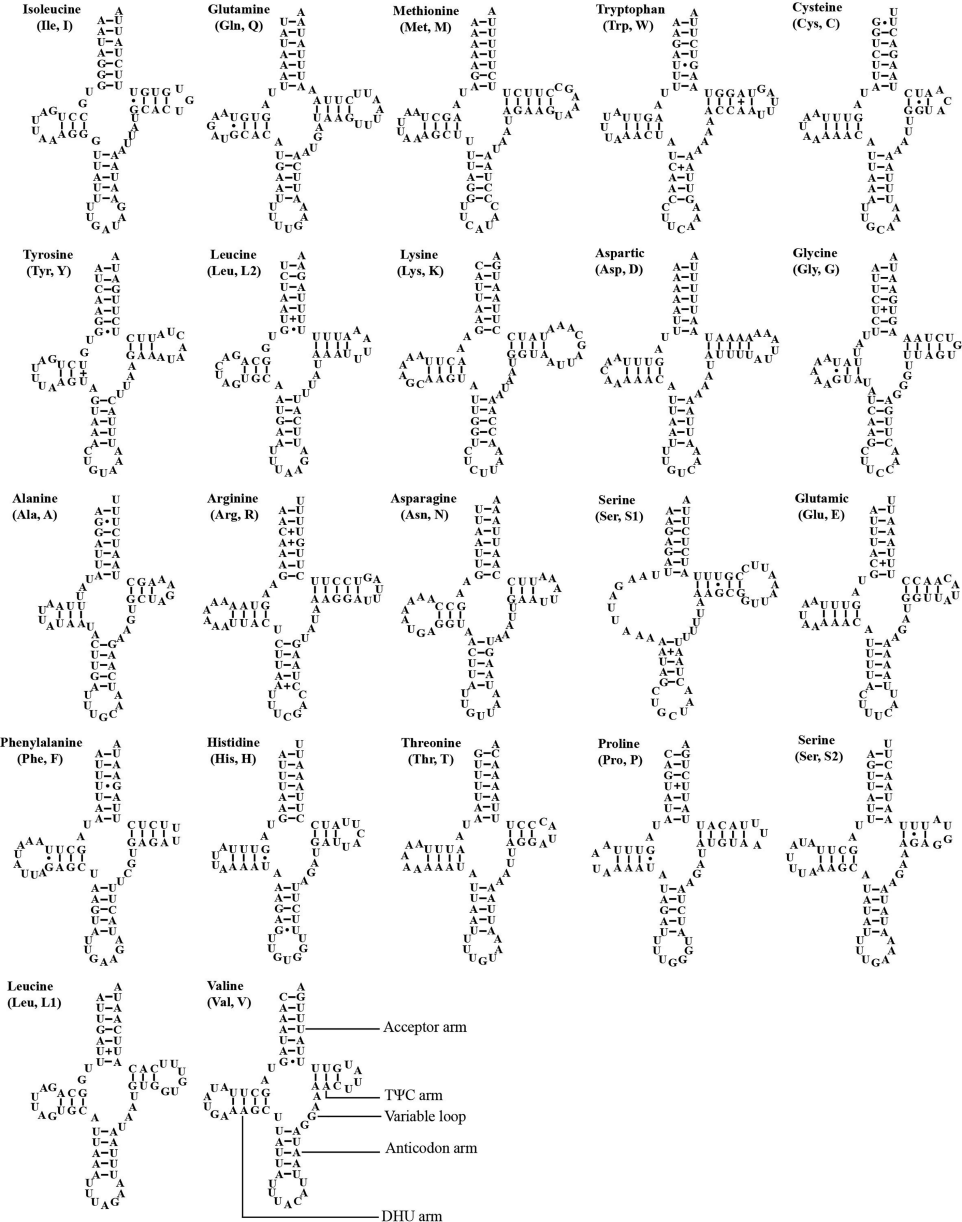
Predicted secondary cloverleaf structure for the tRNAs of *Atkinsoniella zizhongi* sp. nov. mitogenome. The tRNA arms are illustrated as for trnV. Dashes (–), solid dots (•), and pluses (+) indicate the Watson–Crick base pairings, G–U bonds, and mismatches, respectively.

The mitogenome of *A. zizhongi* sp. nov. contained the typical 22 tRNAs, 14 of which were situated on the J-strand, and eight were positioned on the N-strand, with sizes ranging from 61 bp (*trnA* and *trnC*) to 71 bp (*trnK*) (Table 1). The total length of the tRNAs was 1,424 bp, with an AT content of 78.0% (A: 39.7%, T: 38.3%, C: 9.3%, G: 12.7%). The AT skew and GC skew were both positive, with values of 0.017 and 0.157, respectively, suggesting a slight bias toward the use of As and Gs. Most of the tRNAs were predicted to be folded into typical clover-leaf secondary structures, with the exception of trnS1, which lacked the recognizable dihydrouracil (DHU) arm and was replaced with a simple loop (Fig. 4). In the predicted secondary structures, the lengths of the DHU and TΨC loops were variable, resulting in differences in the length of each tRNA. The anticodon loop was highly conserved for 7 bp. In addition, a total of 29 noncanonical base pairs were observed in the 22 tRNAs, including 18 G-U pairs, two A-A pairs, two A-C pairs, five U-U pairs and two U-C pairs.

Two rRNA genes, *l-rRNA* and *s-rRNA*, were recognized in the *A. zizhongi* sp. nov. mitogenome. The *l-rRNA* was 1,205 bp in size and located between *trnL1* and *trnV*; the *s-rRNA* was 810 bp and was found between *trnV* and control region. Both of them were encoded on the N-strand (Fig. 3). The AT content of the rRNAs was 79.6%, with a nucleotide composition of A = 33.3%, T = 46.3%, C = 8.3%, and G = 12.1%. Additionally, the two rRNAs showed a negative AT skew (−0.162) and a positive GC skew (0.187) (Table 2).

### Control regions

The largest noncoding region in the mitogenomes was the control region (also known as the A+T-rich region), which contains the origin of replication and transcription (Cameron, 2014). The control region of *A. zizhongi* sp. nov. mitogenome was typically located between the *s-rRNA* and *trnI* genes and was 2,110 bp in size (Table 1, Fig. 3). The AT content was 71.7%, and both the AT skew and GC skew were positive, with values of 0.035 and 0.308, respectively, indicating that A and G were more abundant than T and C. In this study, two types of tandem repeat units were found in the control region, namely 148 bp with nine repeats and 83 bp with two repeats, respectively (Fig. 3). Furthermore, two poly-T regions with lengths of 25 and 10 bp were also observed.

### Nucleotide diversity and evolutionary rate analysis

Sliding window analysis was conducted to study the nucleotide diversity among the 13 aligned PCGs of the 29 Cicadellinae mitogenomes in this study (Fig. S2). Nucleotide diversity analysis is helpful for identifying high nucleotide divergence regions and for designing species–species markers (Jia et al., 2010; Ma et al., 2020). Our results showed that the nucleotide diversities (Pi values) of different PCGs varied from 0.167 (*COX1*) to 0.347 (*ATP8*). *ATP8*, *ND2*, and *ND4* presented relatively high Pi values of 0.347, 0.274, and 0.224, respectively. The Pi values of *COX1*, *COX2* and *COX3* were comparatively low, at 0.167, 0.172 and 0.177, respectively (Fig. S2), indicating that they were relatively conserved genes within the 13 PCGs. Similar results were produced in the pairwise genetic distance analysis. The largest distance was presented in ATP8, with a value of 0.407, followed by *ND2*, *ND4* and *ATP6*, with values of 0.360, 0.273 and 0.266, respectively. *COX1* possessed the shortest genetic distance (0.191), followed by *COX2* (0.202), *COX3* (0.205) and *ND1* (0.216).

To evaluate the evolutionary rate of PCGs, the ratio of Ka and Ks was estimated for each PCG among these Cicadellinae species (Fig. S3). The Ka/Ks values were all greater than zero and less than one, ranging from 0.067 to 0.716, indicating that all of the PCGs were under purifying selection. Among the 13 PCGs, *COX1* exhibited the strongest purifying pressure with the lowest Ka/Ks value of 0.067. In contrast, *ATP8* was under the weakest purifying pressure and exhibited the highest Ka/Ks value of 0.716. These results are consistent with previous studies of Cicadellinae (Jiang et al., 2022) and other leafhopper subfamilies (Huang & Zhang, 2020; Tang, Huang & Zhang, 2020; Xu, Yu & Zhang, 2020; Lin, Huang & Zhang, 2021; Yu & Zhang, 2021), indicating that *ATP8* may be an ideal barcode gene for species delimitation of Cicadellidae.

### Phylogenetic analysis

Phylogenetic relationships among 29 species of the subfamily Cicadellinae and two outgroups from Mileewinae and Typhlocybinae were reconstructed based on the concatenated datasets of 13 PCGs and two rRNAs. ML and BI analyses were conducted under the best partitioning scheme and models selected by Modelfinder (Table S1) and PartitionFinder (Table S2), respectively. The topological structures presented in the six obtained phylogenetic trees were completely identical, with most nodes receiving high support values in both the ML and BI trees, except for the differences observed in the branches of *B*. *ferruginea*, *B*. *shuana*, and *B*. *qiongana*. In the ML and BI analyses, *B*. *ferruginea* and *B*. *shuana* clustered into a branch, emerging as sister groups with *B*. *qiongana*, except that the phylogenetic relationship of ((*B*. *qiongana* + *B*. *shuana*) + *B*. *ferruginea*) was formed in the cds_faa_ML analysis. Furthermore, the genera *Atkinsoniella* and *Bothrogonia* were strongly supported as monophyletic groups, and the intergeneric relationship within Cicadellinae was consistently recovered as ((((*Atkinsoniella* + *Anagonalia*) + *Gunungidia*) + (*Bothrogonia* + *Kolla*)) + ((*Cicadella* + *Cofana*) + *Homalodisca*)) in all of the phylogenetic trees.

**Figure 5:**
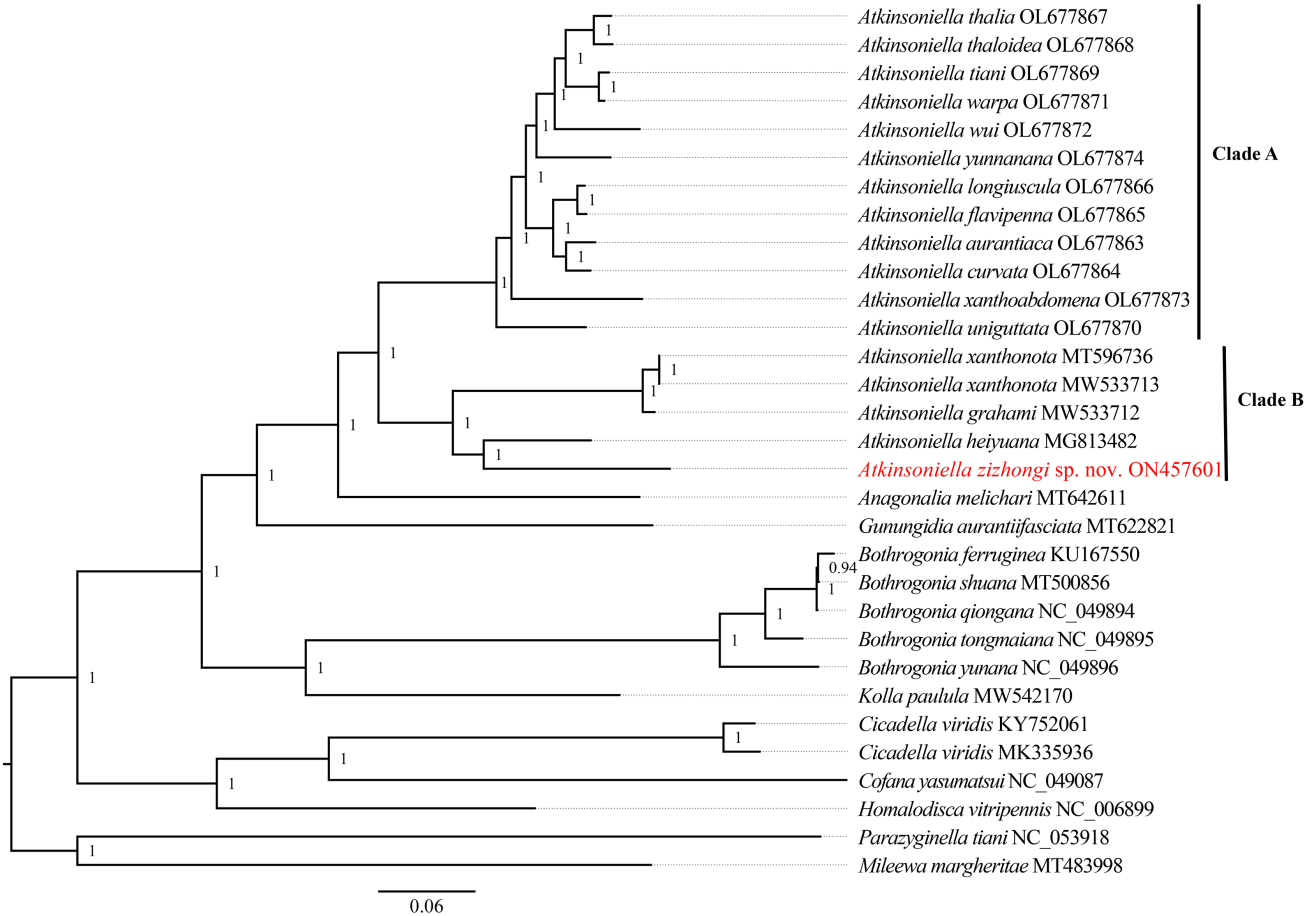
Phylogenetic trees inferred by Bayesian inference (BI) based on the amino acid sequence dataset (cds_faa). Numbers on each node are the posterior probabilities (PP).

In this study, the interspecific relationship of *Atkinsoniella* was the same in all phylogenetic trees, while the relationships among *A*. *yunnanana*, *A*. *uniguttata*, and *A*. *xanthoabdomena* were discrepant across analyses in a previous study (Jiang et al., 2022), which may be related to the addition of new taxa (Havird & Miyamoto, 2010). Additionally, the 16 *Atkinsoniella* species (all mitogenomes of *Atkinsoniella* that have been released on GenBank) were separated into two branches (Clade A and Clade B in Figs. 5, S4–S8). Clade A consisted of 12 yellow-winged *Atkinsoniella* species, and Clade B consisted of four nonyellow-winged species, which was identical to the study of Jiang et al. (2022). Moreover, *A*. *zizhongi* sp. nov. and *A*. *heiyuana* clustered into a branch, forming a sister group with the clade of *A*. *xanthonota* and *A*. *grahami* in all of the resulting trees.

## Conclusion

In this study, a new species *A*. *zizhongi* sp. nov., which is similar to *A*. *nigrominiatula*, *A*. *limba*, *A*. *dormana*, *A*. *peaka*, and *A*. *divaricata* was described. As the identification of Cicadellinae species is mainly based on the external morphological and male genitalia characteristics of adults, female genitalia of different species show little difference, so the females with the similar external morphological characteristics cannot be identified to species level accurately. In view of this, only males of the *A*. *zizhongi* sp. nov. were examined. In addition, the complete mitogenome of *A*. *zizhongi* sp. nov. was determined and analyzed. The obtained six phylogenetic trees suggested that the intergeneric relationships within subfamily Cicadellinae were concurrent and the species of *Atkinsoniella* were consistently separated into two clades across different analyses. Comparing the morphological characteristics of all of the *Atkinsoniella* species in this study, it was obvious that the species situated on the two branches showed significant differences in their external morphological characteristics, including color and markings on the crown and face. Overall, the results of the phylogenetic analyses were highly congruent with the preliminary grouping based on their morphological characteristics. Therefore, mitogenome can be considered as an effective way to help clarify the intergeneric and interspecific relationships. However, the lack of Cicadellinae mitogenomes limits the studies of the phylogeny within the subfamily. Hence, it is necessary to obtain more molecular data of Cicadellinae species from other geographical regions to help us elucidate the phylogenetic relationships of Cicadellinae and higher taxa.

## Supplemental Information

10.7717/peerj.14026/supp-1 Supplemental Information 1 The complete mitochondrial genome sequence of *Atkinsoniella zizhongi* sp. nov.

10.7717/peerj.14026/supp-2 Supplemental Information 2 The complete mitochondrial genome sequence of *Atkinsoniella zizhongi* sp. nov.

10.7717/peerj.14026/supp-3 Supplemental Information 3 Best models were calculated by Modelfinder of cds_faa, cds12_fna, cds12_rrna datasets used in analysis. cds_faa: amino acid sequences of the protein-coding genes (PCGs); cds12_fna: first and second codon positions of PCGs; cds12_rrna: the first and the second codon positions of the PCGs and two rRNA genes.

10.7717/peerj.14026/supp-4 Supplemental Information 4 Best models were calculated by PartitionFinder2 of cds_faa, cds12_fna, cds12_rrna datasets used in analysis. cds_faa: amino acid sequences of the protein-coding genes (PCGs); cds12_fna: first and second codon positions of PCGs; cds12_rrna: the first and the second codon positions of the PCGs and two rRNA genes.

10.7717/peerj.14026/supp-5 Supplemental Information 5 The codon number and relative synonymous codon usage (RSCU) of PCGs in*Atkinsoniella zizhongi*sp. nov. mitogenome. A: The codon number of PCGs in*A. zizhongi*sp. nov. mitogenome. B: The relative synonymous codon usage (RSCU) of PCGs in*A. zizhongi*sp. nov. mitogenome. The codons of each family are shown in colored boxes below the x-axis, and the number of amino acids and RSCU values are displayed on the y-axis. The colors correspond to the stacked columns.

10.7717/peerj.14026/supp-6 Supplemental Information 6 Sliding window analysis based on the 13 aligned PCGs. The red line shows the nucleotide diversity Pi value (window size = 200 bp, step size = 20 bp).

10.7717/peerj.14026/supp-7 Supplemental Information 7 The ratio of nonsynonymous (Ka) to synonymous (Ks) substitution rates and the average genetic distance of 13 PCGs among 29 Cicadellinae species.

10.7717/peerj.14026/supp-8 Supplemental Information 8 Phylogenetic tree inferred by maximum likelihood (ML) based on the amino acids (cds_faa). Numbers on each node are bootstrap support values (BS).

10.7717/peerj.14026/supp-9 Supplemental Information 9 Phylogenetic tree inferred by maximum likelihood (ML) based on the 1st and 2nd code position of protein-coding genes (cds12_fna). Numbers on each node correspond to the bootstrap support values (BS).

10.7717/peerj.14026/supp-10 Supplemental Information 10 Phylogenetic tree inferred by Bayesian inference (BI) based on the 1st and 2nd code position of protein-coding genes (cds12_fna). Numbers on each node are the posterior probabilities (PP).

10.7717/peerj.14026/supp-11 Supplemental Information 11 Phylogenetic tree inferred by maximum likelihood (ML) based on the 1st and 2nd code position of protein coding genes concatenated rRNA genes (cds12_rrna). Numbers on each node are the bootstrap support values (BS).

10.7717/peerj.14026/supp-12 Supplemental Information 12 Phylogenetic tree inferred by Bayesian inference (BI) based on the 1st and 2nd code position of protein-coding genes concatenated rRNA genes (cds12_rrna). Numbers on each node are the posterior probabilities (PP).

## Additional Information and Declarations

### Field Study Permissions

The following information was supplied relating to field study approvals (*i.e*., approving body and any reference numbers): The field approval documents for collecting specimens are provided in Supplemental Files 1 and 2.

### Data Availability

The following information was supplied regarding data availability: The complete mitogenome sequence of Atkinsoniella sp. nov. are both available in the Supplemental Files and at Genbank: ON457601.

### New Species Registration

The following information was supplied regarding the registration of a newly described species: *Atkinsoniella zizhongi* sp. nov LSID: urn:lsid:zoobank.org:act:AE46396A-3295-4284-9830-60FEB4398BF9. Publication LSID: urn:lsid:zoobank.org:pub:0179516A-96AA-4356-81A4-23C5D3259C4C.
