# Supplementary material for: Description and complete mitochondrial genome of Atkinsoniella zizhongi sp. nov. (Hemiptera: Cicadellidae: Cicadellinae) from China and its phylogenetic implications
Source: PeerJ. 2022 Sep 28;10:e14026. doi: 10.7717/peerj.14026 (PMC9526418; doi:10.7717/peerj.14026)
Supplement: Supplemental Information 4 — cds_faa: amino acid sequences of the protein-coding genes (PCGs); cds12_fna: first and second codon positions of PCGs; cds12_rrna: the first and the second codon positions of the PCGs and two rRNA genes. [file peerj-10-14026-s004.docx]

|  | **Table S2.** Best models were calculated by PartitionFinder2 of cds_faa, cds12_fna, cds12_rrna datasets used in analysis. | | | |
| --- | --- | --- | --- | --- |
| **Dataset** | **starting partitions** | **Subset** | **Subset Partitions** | **Best Model** |
| cds_faa | ATP6 = 1-217; ATP8 = 218-252; COX1 = 253-763; COX2 = 764-989; COX3 = 990-1248; CYTB = 1249-1626; ND1 = 1627-1936; ND2 = 1937-2256; ND3 = 2257-2372; ND4L = 2373-2465; ND4 = 2466-2905; ND5 = 2906-3459; ND6 = 3460-3612; | P1 | ND5, ATP6, ATP8, ND4 | MTREV+I+G |
|  |  | P2 | COX1 | MTMAM+I+G |
|  |  | P3 | COX2, CYTB, COX3, | MTREV+I+G |
|  |  | P4 | ND4L, ND1 | MTMAM+G |
|  |  | P5 | ND2 | MTREV+G |
|  |  | P6 | ND3, ND6 | MTREV+G |
| cds12_fna | COX1=1-1024; ND1=1025-1646; COX2=1647-2098; ND2=2099-2746; COX3=2747-3266; ND3=3267-3498; ND4=3499-4380; ND5=4381-5496; ATP6=5497-5932; ND6=5933-6248; ATP8=6249-6350; CYTB=6351-7108; ND4L=7109-7296; | P1 | COX1, CYTB, COX3, COX2 | GTR+I+G |
|  |  | P2 | ND1, ND4, ND4L, ND5 | GTR+I+G |
|  |  | P3 | ND2, ATP8 | GTR+I+G |
|  |  | P4 | ATP6, ND6, ND3 | GTR+I+G |
| cds12_rrna | COX1=1-1024; ND1=1025-1646; COX2=1647-2098; ND2=2099-2746; COX3=2747-3266; ND3=3267-3498; ND4=3499-4380; ND5=4381-5496; ATP6=5497-5932; ND6=5933-6248; ATP8=6249-6350; CYTB=6351-7108; ND4L=7109-7296; l-rRNA = 7297-8403; s-rRNA = 8404-9065; | P1 | COX1, CYTB, COX3, COX2 | GTR+I+G |
|  |  | P2 | ND1, ND4, ND4L, ND5 | GTR+I+G |
|  |  | P3 | ND2, ATP8 | GTR+I+G |
|  |  | P4 | ATP6, ND6, ND3 | GTR+I+G |
|  |  | P5 | l-rRNA, s-rRNA | GTR+G |

cds_faa: amino acid sequences of the protein-coding genes (PCGs); cds12_fna: first and second codon positions of PCGs; cds12_rrna: the first and the second codon positions of the PCGs and two rRNA genes.
