# Supplementary figures and images for: Description and complete mitochondrial genome of Atkinsoniella zizhongi sp. nov. (Hemiptera: Cicadellidae: Cicadellinae) from China and its phylogenetic implications
Source: PeerJ. 2022 Sep 28;10:e14026. doi: 10.7717/peerj.14026 (PMC9526418; doi:10.7717/peerj.14026)

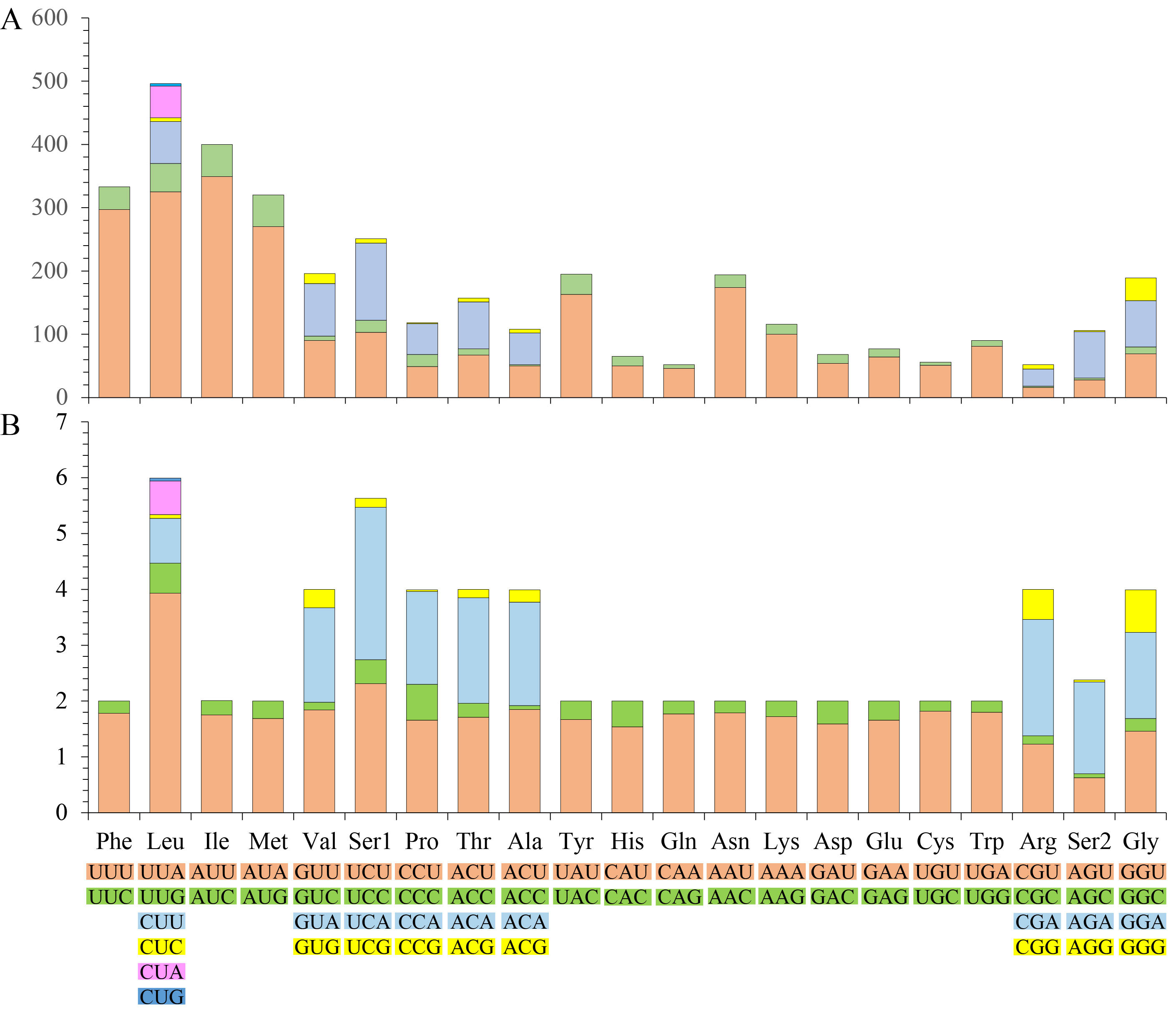

Supplement: Supplemental Information 5 — A: The codon number of PCGs inA. zizhongisp. nov. mitogenome. B: The relative synonymous codon usage (RSCU) of PCGs inA. zizhongisp. nov. mitogenome. The codons of each family are shown in colored boxes below the x-axis, and the number of amino acids and RSCU values are displayed on the y-axis. The colors correspond to the stacked columns. [file peerj-10-14026-s005.jpg]

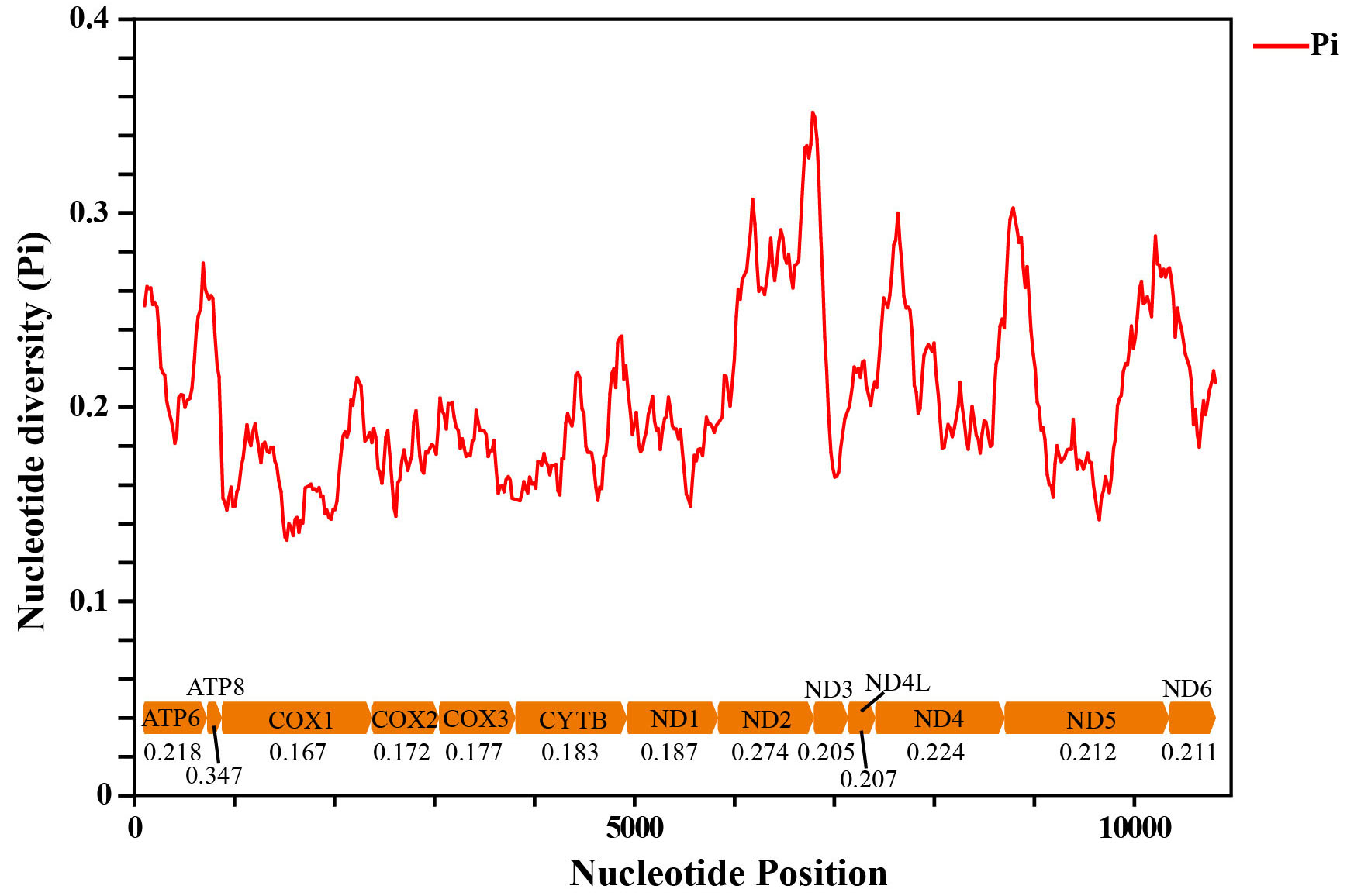

Supplement: Supplemental Information 6 [file peerj-10-14026-s006.jpg]

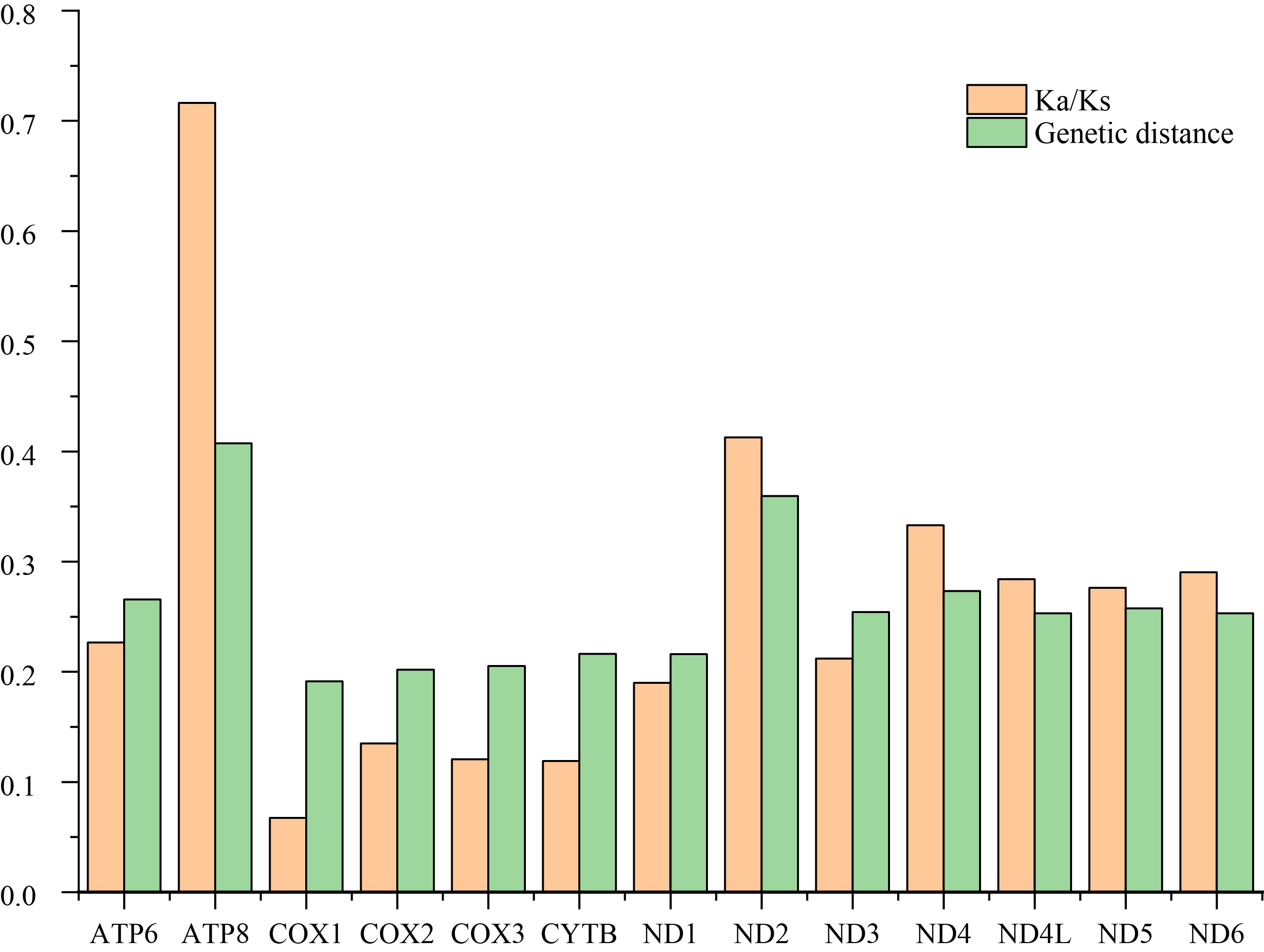

Supplement: Supplemental Information 7 [file peerj-10-14026-s007.jpg]

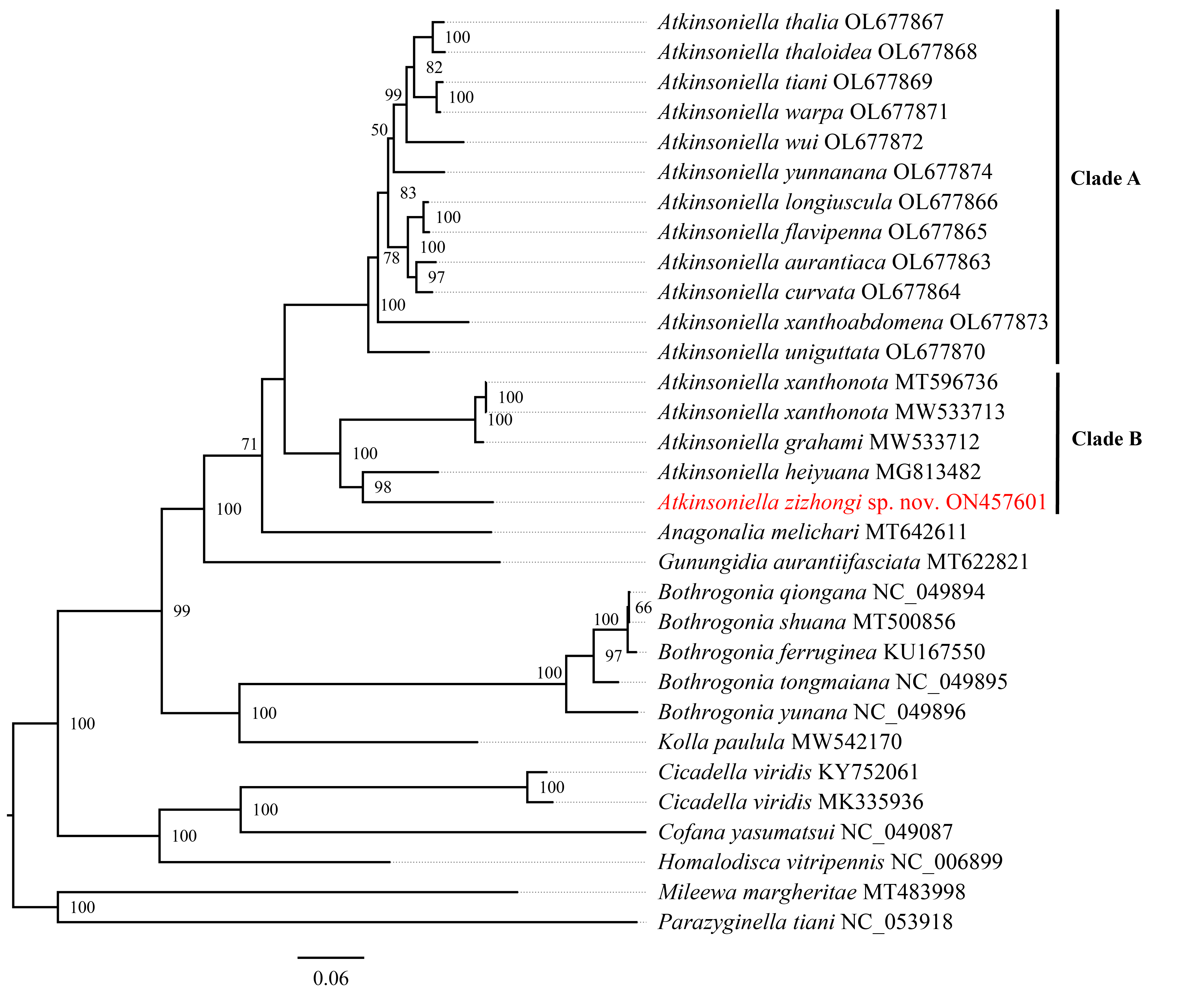

Supplement: Supplemental Information 8 — Numbers on each node are bootstrap support values (BS). [file peerj-10-14026-s008.jpg]

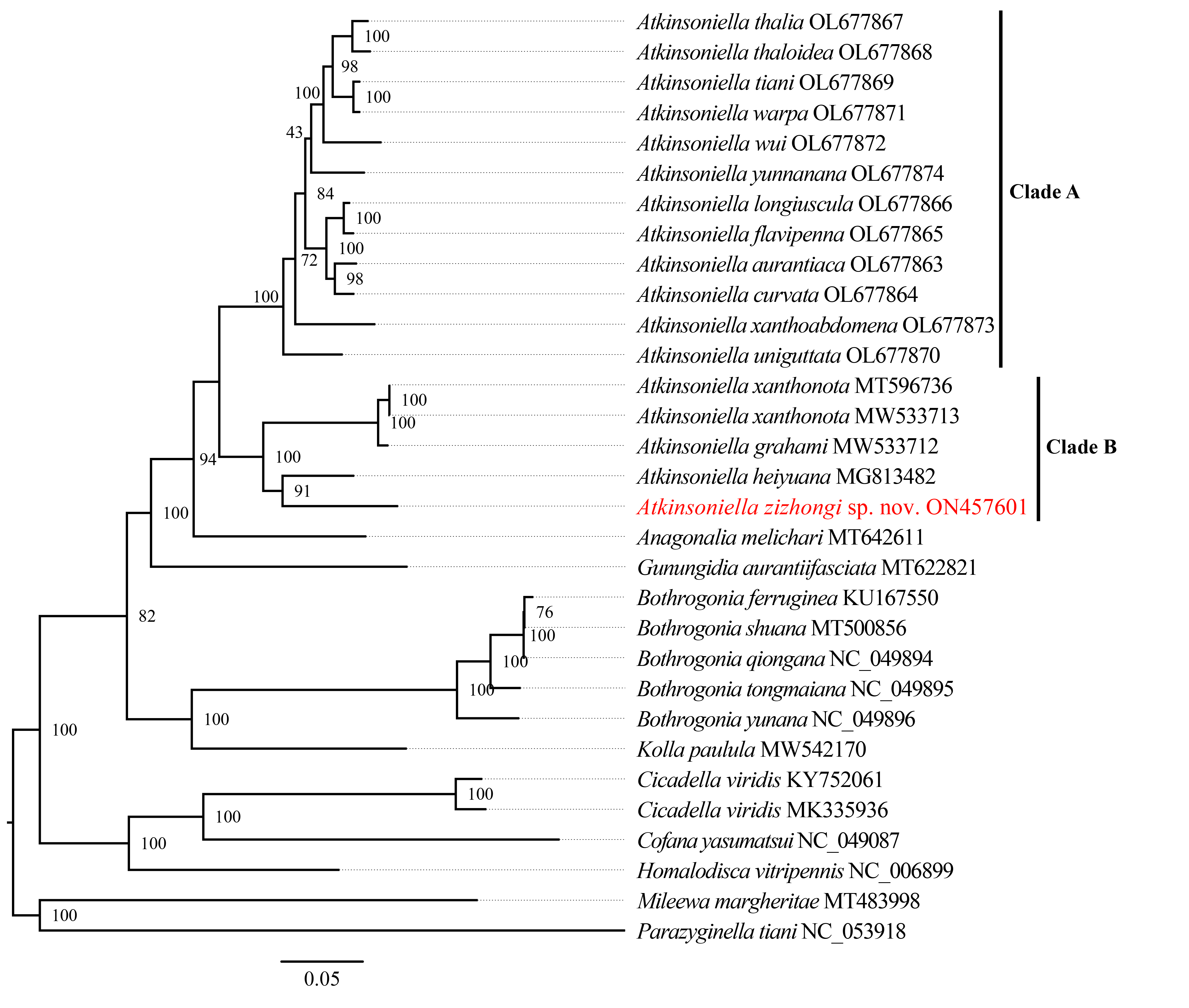

Supplement: Supplemental Information 9 — Numbers on each node correspond to the bootstrap support values (BS). [file peerj-10-14026-s009.jpg]

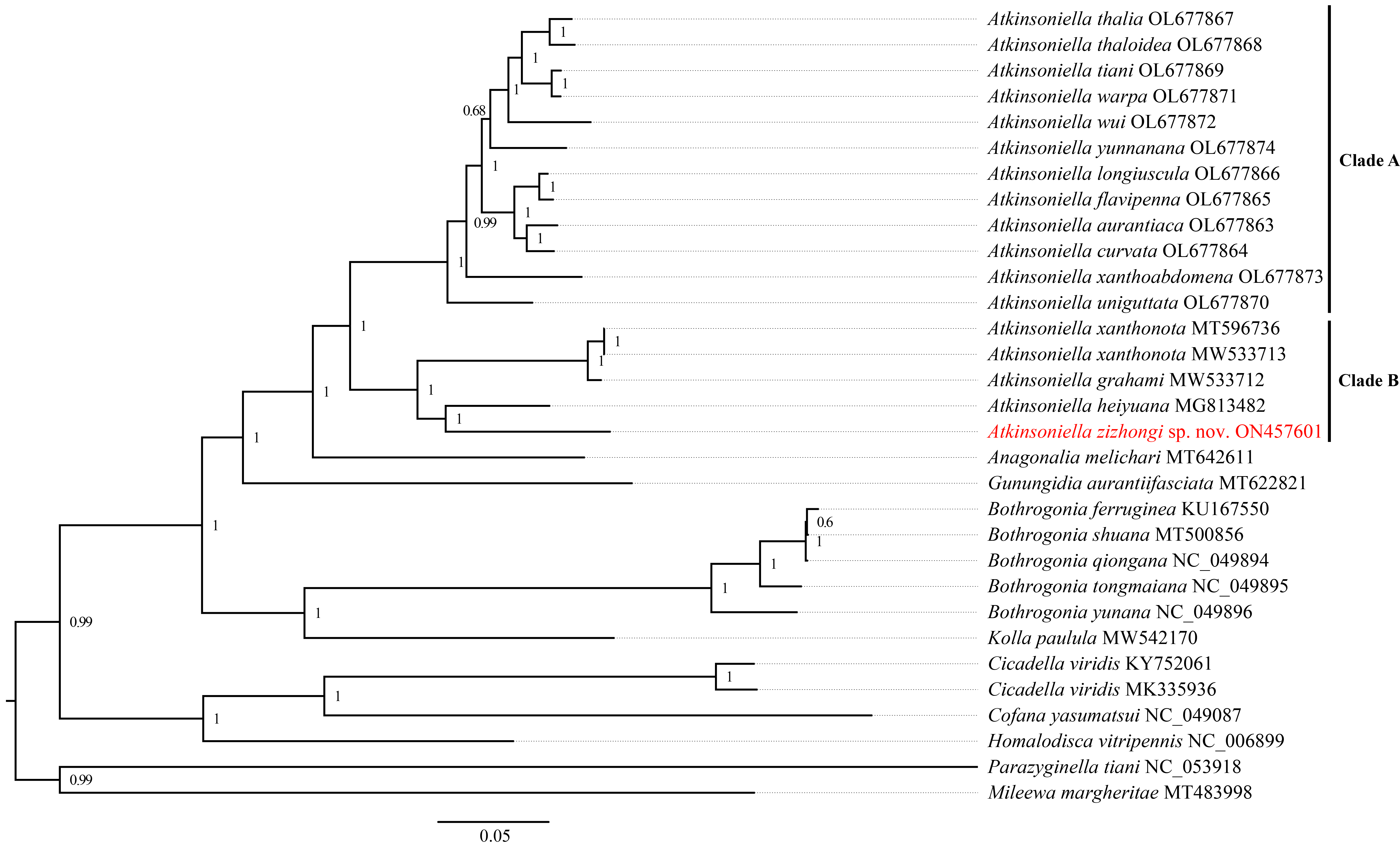

Supplement: Supplemental Information 10 — Numbers on each node are the posterior probabilities (PP). [file peerj-10-14026-s010.jpg]

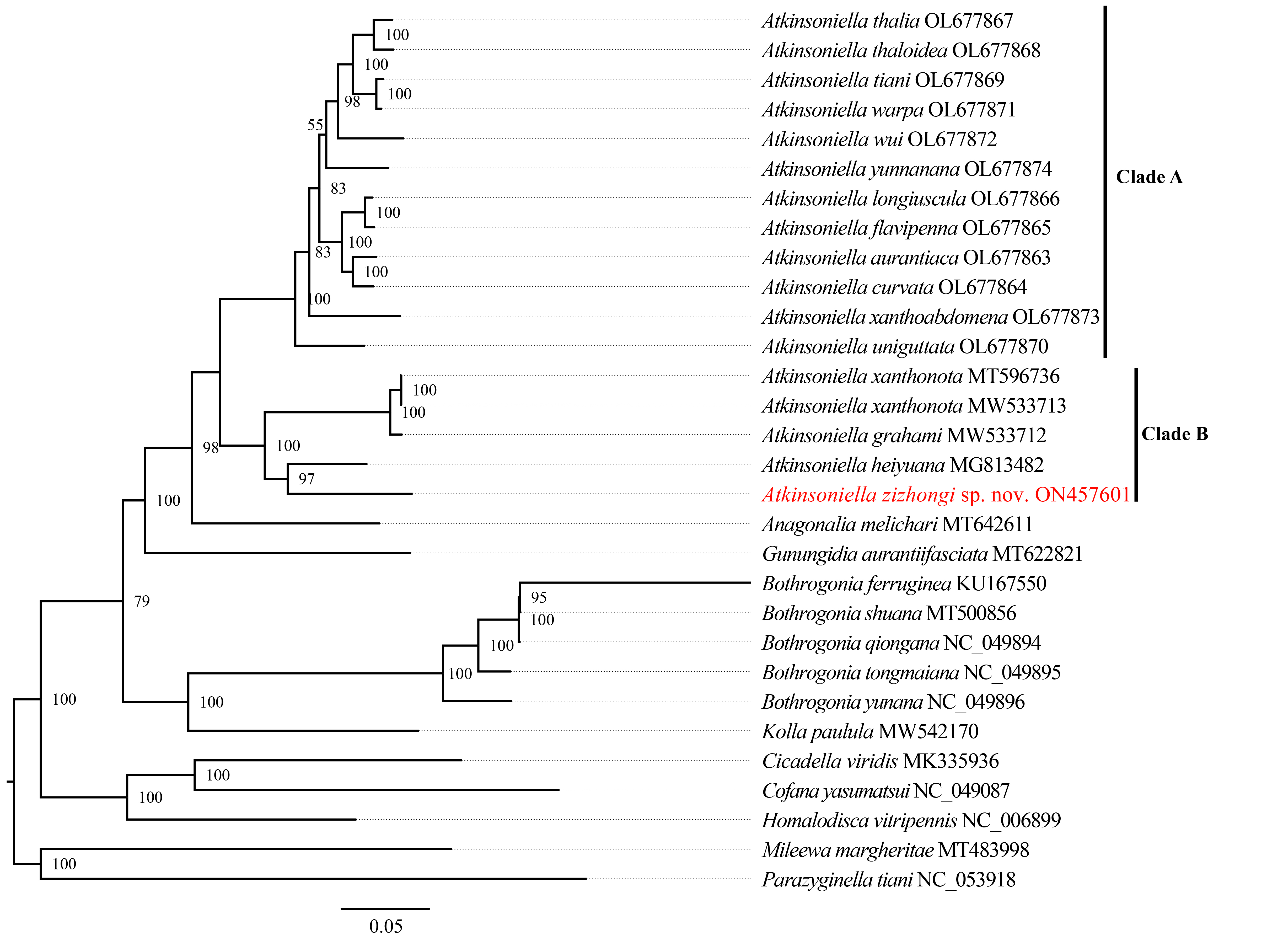

Supplement: Supplemental Information 11 — Numbers on each node are the bootstrap support values (BS). [file peerj-10-14026-s011.jpg]

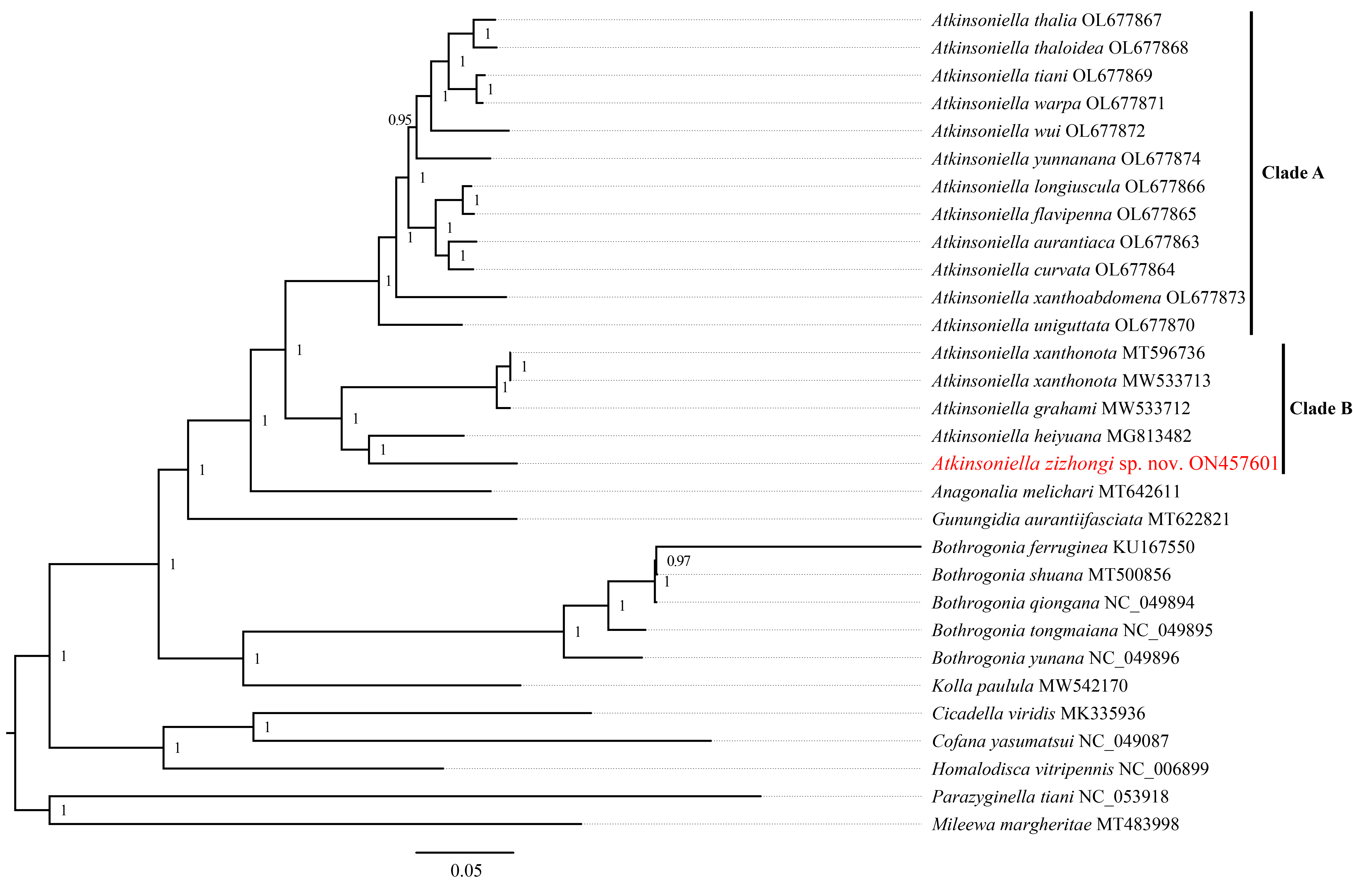

Supplement: Supplemental Information 12 — Numbers on each node are the posterior probabilities (PP). [file peerj-10-14026-s012.jpg]
